# Buthalital and methitural – 5,5-substituted derivatives of 2-thio­barbituric acid forming the same type of hydrogen-bonded chain

**DOI:** 10.1107/S205698901701653X

**Published:** 2017-11-21

**Authors:** Thomas Gelbrich, Ulrich J. Griesser

**Affiliations:** a University of Innsbruck, Institute of Pharmacy, Innrain 52, 6020 Innsbruck, Austria

**Keywords:** crystal structure, hydrogen bonding, barbiturates, pharmaceuticals

## Abstract

The mol­ecule of buthalital, (I) [systematic name: 5-(2-methyl­prop­yl)-5-(prop-2-en-1-yl)-2-sulfanyl­idene-1,3-diazinane-4,6-dione], C_11_H_16_N_2_O_2_S, exhibits a planar pyrimidine ring, whereas the pyrimidine ring of methitural, (II) [systematic name: 5-(1-methyl­but­yl)-5-[2-(methyl­sulfan­yl)eth­yl]-2-sulfanyl­idene-1,3-diazinane-4,6-dione], C_12_H_20_N_2_O_2_S_2_, is slightly puckered. (I) and (II) contain the same hydrogen-bonded chain structure in which each mol­ecule is connected, *via* four N—H⋯O=C hydrogen bonds, to two other mol­ecules, resulting in a hydrogen-bonded chain displaying a sequence of *R*_2_^2^(8) rings. The same type of N—H⋯O=C hydrogen-bonded chain has previously been found in several 5,5-disubstituted derivatives of barbituric acid which are chemically closely related to (I) and (II).

## Abstract 2

In the title structures, each mol­ecules is connected to two other mol­ecules *via* four N—H⋯O hydrogen bonds, resulting in a chain with a sequence of 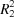
(8) rings.

## Chemical context   

Buthalital (I) and methitural (II) are 5,5-disubstituted derivatives of 2-thio­barbituric acid. Compounds of the thio­barbiturate class differ from the corresponding barbiturates in that the ketone group at the 2-position is replaced by a thione group. Thio­barbiturates are used as injection narcotics for the induction of general anaesthesia or to produce complete anaesthesia of short duration. The sodium salt of (I) was originally developed as a short-acting anaesthetic but was found to have an extremely rapid elimination rate. Similarly, (II) was marketed in the 1950s as an ultra-short-acting intra­venous anaesthetic.
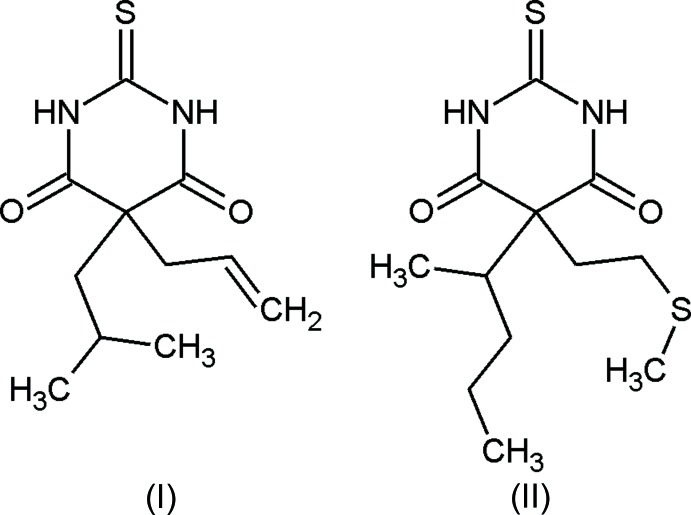


## Structural commentary   

**Figure 1:**
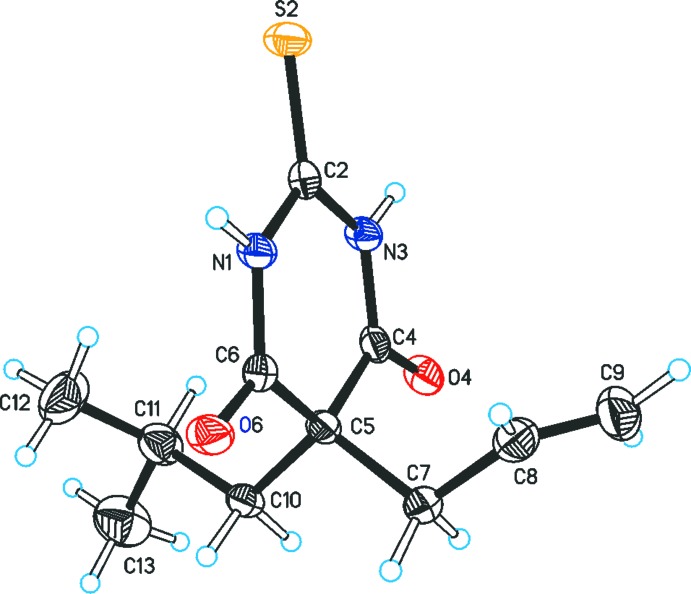
The mol­ecular structure of (I), with displacement ellipsoids drawn at the 50% probability level and H atoms drawn as spheres of arbitrary size.

The mol­ecular structure of (I), Fig. 1 ▸, shows an almost planar pyrim­idine ring (N1, C2, N3, C4 C5, C6) with a root-mean-square (r.m.s.) deviation of its six atoms from the mean plane of 0.016 Å (Fig. 1 ▸). The (C7, C8, C5, C10, C11) unit defined by ring atom C5 and two atoms of each of the allyl and isobutyl substituents is nearly planar (r.m.s. deviation = 0.050 Å). The mean plane of this fragment forms an angle of 87.5 (1)° with the plane of the six-membered ring. Additionally, it forms an angle of 77.8 (2)° with the plane of the allyl group defined by C7, C8 and C9. The terminal torsion angles C5—C10—C11—C12 and C5—C10— C11—C13 of the isobutyl substituent are −71.7 (3)° and 165.6 (2)°, respectively.

**Figure 2:**
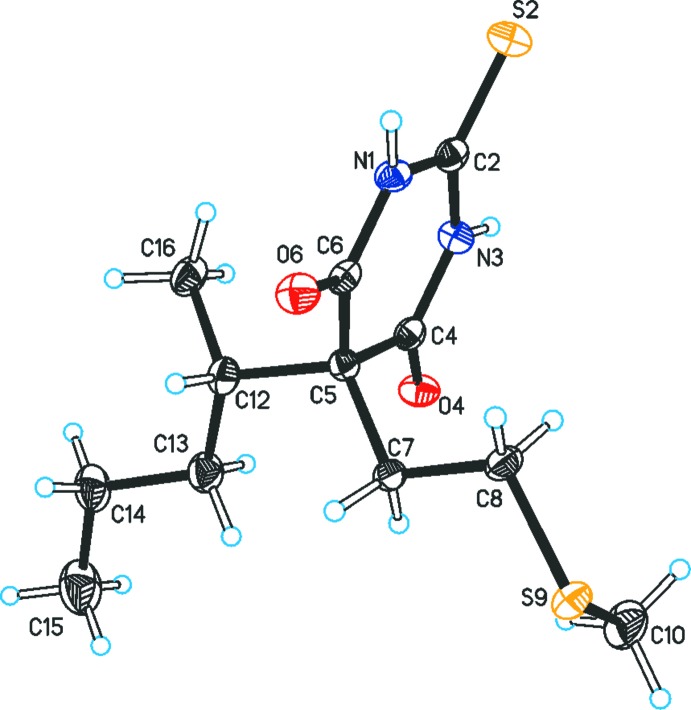
The mol­ecular structure of (II), with displacement ellipsoids drawn at the 50% probability level and H atoms drawn as spheres of arbitrary size.

The pyrimidine ring (N1, C2, N3, C4 C5, C6) in the mol­ecule of (II) deviates somewhat from planarity (r.m.s. deviation = 0.030 Å); specifically, the distance between C6 and the mean plane defined by the other five ring atoms (r.m.s deviation = 0.005 Å) is 0.104 (2) Å (Fig. 2 ▸). The mean plane of the (S9, C8, C7, C5, C12, C16) chain, defined by ring atom C6, three atoms of the 2-(methyl­thio)­ethyl substituent and two atoms of the *sec*-butyl group (r.m.s. deviation = 0.091 Å) forms an angle of 88.64 (5)° with the mean plane of the pyrimidine ring and an angle of 39.0 (1)° with the mean plane of the (C5, C12, C13, C14, C15) fragment of the nearly planar (r.m.s. deviation = 0.070) *sec*-butyl group. In the 2-(methyl­thio)­ethyl substituent, the C10—S9 and C8—S9 bond lengths are 1.794 (2) and 1.803 (2) Å, respectively, and the C7—C8—S9—C10 torsion angle is 82.5 (2)°. The bond between ring atom C5 and atom C12 of the *sec*-butyl group [1.582 (2) Å] is somewhat longer than the analogous distance between C5 and atom C7 of the 2-(methyl­thio)­ethyl group [1.547 (2) Å]. This difference is reminiscent of the difference between equatorial and axial bonds at ring atom C5 found in several 5,5-disubstituted barbituric acid derivatives that exhibit a puckered pyrimidine ring (Gelbrich *et al.*, 2016*b* ▸).

## Supra­molecular features   

**Figure 3:**
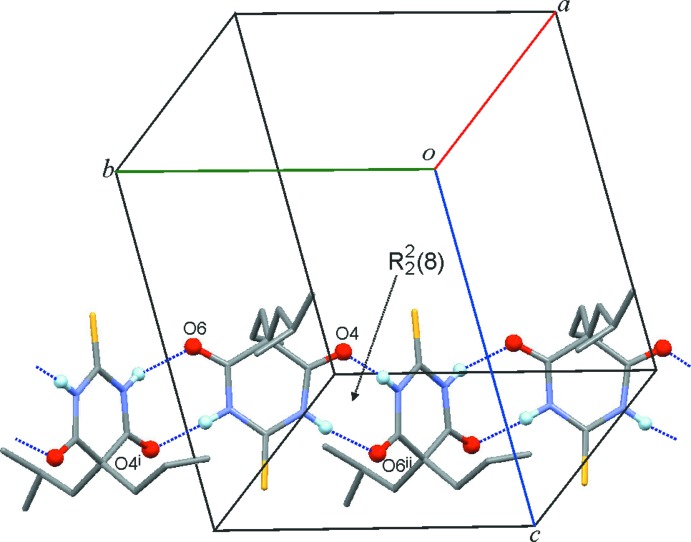
The **C-2**-type bonded chain of (I). O and H atoms directly involved in N—H⋯O inter­actions are drawn as balls and H atoms bonded to C atoms are omitted for clarity. The chain displays a twofold screw symmetry and contains just one type of 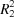
(8) ring. [Symmetry codes: (i) −*x* + 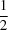, *y* + 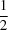, −*z* + 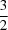; (ii) −*x* + 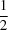, *y* − 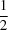, −*z* + 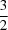.]

The crystal structure of (I) contains N1—H1⋯O4^i^ and N3—H3⋯O6^ii^ bonds (Fig. 3 ▸, Table 1 ▸). Each mol­ecule is linked to two neighbouring mol­ecules *via* two-point connections and 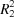
(8) rings (Etter *et al.* 1990 ▸, Bernstein *et al.*, 1995 ▸). The resulting chain structure (topological type 2C1) contains a twofold screw axis and runs parallel to the *b* axis. The mean planes of neighbouring pyrimidine rings in the chain form an angle of approximately 40° with one another. The chain structure of (I) belongs to the **C-2** type, which also occurs in a number of 5,5-disubstituted barbituric acid derivatives (Gelbrich *et al.*, 2016*a* ▸). The four shortest inter­molecular contacts of the sulfur atom (S⋯H distances between 2.97 and 3.01 Å; close to the sum of van der Waals radii) involve both CH_2_ groups of a neighbouring mol­ecule and one CH_3_ group belonging to the isobutyl substituent of two other mol­ecules.

**Figure 4:**
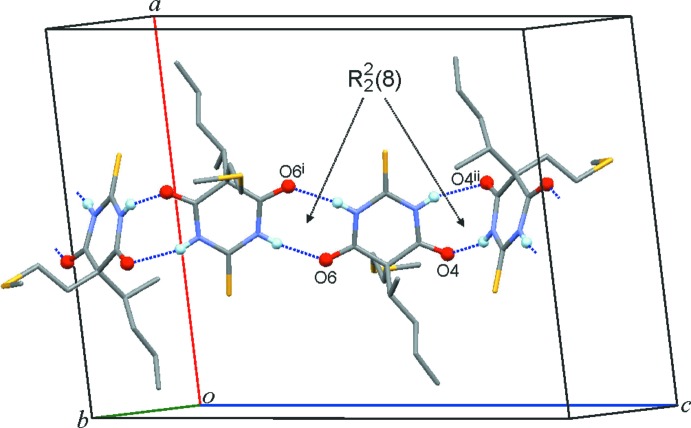
The **C**-**2**-type bonded chain of (II). O and H atoms directly involved in N—H⋯O inter­actions are drawn as balls and H atoms bonded to C atoms are omitted for clarity. The chain displays two types of 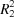
(8) ring, which contain an inversion centre (N1—H1⋯O6^i^) or a twofold axis (N3—H3⋯O4^ii^). [Symmetry codes: (i) −*x*, −*y* + 1, −*z* + 1; (ii) −*x*, *y*, −*z* + 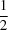.]

Two independent hydrogen bonds, N1—H1⋯O6^i^ and N3—H3⋯O4^ii^, are present in the crystal structure of (II). As in (I), each mol­ecule is linked, by two-point connections, to two neighbouring mol­ecules so that a **C-2** chain structure is formed that propagates parallel to the *c* axis. In this case, the **C-2** chain contains two crystallographically distinct 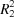
(8) rings which are centred either by a twofold axis or an inversion centre (Fig. 4 ▸, Table 2 ▸). The mean planes of adjacent pyrimidine rings in the same chain are either coplanar with one another (if the corresponding mol­ecules are related by an inversion operation), or they form an angle of 75° (if the mol­ecules are related by a 180° rotation). The sulfur atom S9 of the 2-(methyl­thio)­ethyl substituent forms an inter­molecular contact (S⋯H = 2.86 Å) with the *sec*-butyl group of a mol­ecule belonging to a neighbouring chain and S2 lies in close proximity to the methyl group of a 2-(methyl­thio)­ethyl substituent (S⋯H = 2.96 Å).

## Database survey   

The crystal structures of three polymorphs of the keto form of 2-thio­barbituric acid, which is a close structural analogue of (I) and (II), have been determined (Chierotti *et al.*, 2010 ▸). Polymorph III (CSD refcode THBARB01) contains an N—H⋯O-bonded layer structure having the **hcb** topology and polymorph IV (THBARB02) an N—H⋯O-bonded framework. Both these structures contain N—H⋯O-bonded 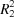
(8) rings analogous to those present in the hydrogen-bonded chains of (I) and (II), and additionally they contain one-point hydrogen-bond connections between mol­ecules. Form VI of 2-thio­barbituric acid (THBARB03) displays two distinct hydrogen-bonded structures, an N—H⋯O-bonded layer with **sql** topology whose mol­ecules are linked exclusively by one-point connections and an **hcb**-type layer based on N—H⋯O as well as N—H⋯S bonds, with the latter inter­action resulting in 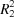
(8) rings.

**Figure 5:**
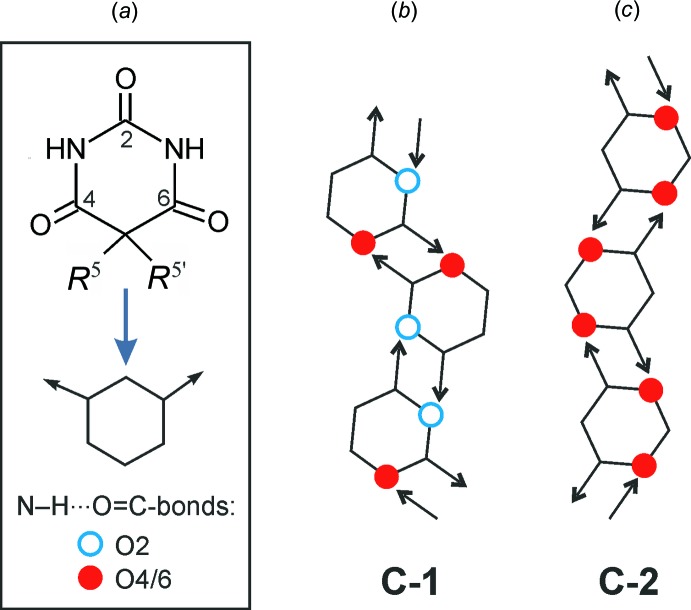
(*a*) Simplified representation of a mol­ecule of a 5,5-disubstituted derivative of barbituric acid. The same scheme can be applied for analogous thio­barbiturates such as (I) and (II) if the O atom of the carbonyl group in position 2 is replaced by a thioxo S atom. (*b*) and (*c*) Schematic representation of the N—H⋯O=C-bonded chain types **C-1** and **C-2** with an underlying 2C1 topology, which are frequently found in barbiturates. The thio­barbiturates (I) and (II) contain chains of the **C**-**2** type.

Numerous 5,5-substituted derivatives of barbituric acid are known to form N—H⋯O=C-bonded chains exhibiting the 2C1 topology, with their mol­ecules being linked by two-point connections resulting in the formation of characteristic 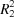
(8) rings. Chains exhibiting these specific properties can be classified into two distinct types, denoted as **C-1** and **C-2** (Gelbrich *et al.*, 2016*a* ▸; see Fig. 5 ▸). The less frequent of these two types, **C-2**, is also the chain motif of (I) and (II). It is characterized by the employment of each of the topologically equivalent C4 and C6 carbonyl groups, but not the C2 group, as a hydrogen-bond acceptor.

**C-2** chains containing a 2_1_ screw axis occur in polymorph III of phenobarbital (PHBARB09), the CH_2_Cl_2_ solvate of the same compound (EPUDEA) (Zencirci *et al.*, 2010 ▸, 2014 ▸) and in 5-fluoro-5-phenyl­barbituric acid (HEKTOG) (DesMarteau *et al.*, 1994 ▸) as well as in (I). By contrast, the **C-2** chains of 6-oxo­cyclo­barbital (OXCBAR) (Chentli-Benchikha *et al.*, 1977 ▸) and polymorph III of pentobarbital (FUFTEG02) (Rossi *et al.*, 2012 ▸) exhibit glide symmetry. Moreover, polymorph II of barbital (DETBAA02) (Craven *et al.*, 1969 ▸) as well as forms I and II of phenobarbital (Zencirci *et al.*, 2010 ▸) exhibit **C-2** chains whose 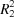
(8) rings contain crystallographic inversion centres. The crystal structure of methitural (II) is the first example of a **C-2** chain whose 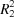
(8) rings are centred alternately by a twofold rotational axis and an inversion centre.

## Synthesis and crystallization   

Single crystals of (I) were produced by sublimation between two glass slides separated by a spacer ring (height: 1 cm), using a hot bench at a temperature of 403 K. As confirmed by PXRD, the phase investigated by us is identical with that of the original sample from the1940s obtained from our archive. The melting point of this phase of 422 K was determined with hot-stage microscopy. Heating the quench-cooled melt of (I) above 323 K resulted in the crystallization of a second form. Isolated, individual crystals of this second form melted at approximately 387 K. In other experiments, a phase transition from the low-melting form II to a high-melting form I occurred on heating, usually between 378 and 383 K (see Supporting information). These observations are consistent with a previous description by Brandstätter-Kuhnert & Aepkers (1962 ▸).

The crystals of (II) investigated in this study were taken from a sample obtained from Merck AG, Darmstadt, Germany. These crystals melted within a relatively broad temperature range between 361 and 366 K.

## Refinement   

Crystal data, data collection and structure refinement details are summarized in Table 3 ▸. All H atoms were identified in difference maps. Methyl H atoms were idealized and included as rigid groups allowed to rotate but not tip and all other H atoms bonded to carbon atoms were positioned geometrically (C—H = 0.95–0.99 Å). The hydrogen atoms in NH groups were refined with restrained distances [N—H = 0.88 (2) Å]. The *U*_iso_ parameters of all H atoms were refined freely.

## Supplementary Material

Crystal structure: contains datablock(s) I, II, global. DOI: 10.1107/S205698901701653X/dx2002sup1.cif

Structure factors: contains datablock(s) I. DOI: 10.1107/S205698901701653X/dx2002Isup2.hkl

Structure factors: contains datablock(s) II. DOI: 10.1107/S205698901701653X/dx2002IIsup3.hkl

Supporting information file. DOI: 10.1107/S205698901701653X/dx2002Isup4.cml

Supporting information file. DOI: 10.1107/S205698901701653X/dx2002IIsup5.cml

Hot-stage microscopy. DOI: 10.1107/S205698901701653X/dx2002sup6.pdf

CCDC references: 1586016, 1586015

Additional supporting information:  crystallographic information; 3D view; checkCIF report

## Figures and Tables

**Table 1:** Hydrogen-bond geometry (Å, °) for (I)

*D*—H⋯*A*	*D*—H	H⋯*A*	*D*⋯*A*	*D*—H⋯*A*
N1—H1⋯O4^i^	0.87 (2)	1.95 (2)	2.815 (2)	174 (2)
N3—H3⋯O6^ii^	0.86 (2)	2.10 (2)	2.922 (2)	160 (2)

Symmetry codes: (i) 
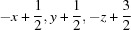
; (ii) 
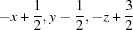
.

**Table 2:** Hydrogen-bond geometry (Å, °) for (II)

*D*—H⋯*A*	*D*—H	H⋯*A*	*D*⋯*A*	*D*—H⋯*A*
N1—H1⋯O6^i^	0.86 (2)	2.07 (2)	2.921 (2)	170 (2)
N3—H3⋯O4^ii^	0.86 (2)	2.14 (2)	2.963 (2)	160 (2)

Symmetry codes: (i) 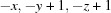; (ii) 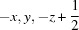.

**Table 3:** Experimental details

	(I)	(II)
Crystal data		
Chemical formula	C_11_H_16_N_2_O_2_S	C_12_H_20_N_2_O_2_S_2_
*M*_r_	240.32	288.42
Crystal system, space group	Monoclinic, *P*2_1_/*n*	Monoclinic, *C*2/*c*
Temperature (K)	120	120
*a*, *b*, *c* (Å)	8.7271 (6), 11.6521 (4), 12.5400 (8)	15.1873 (2), 9.0920 (1), 20.8684 (3)
β (°)	96.539 (2)	96.083 (1)
*V* (Å^3^)	1266.89 (13)	2865.34 (6)
*Z*	4	8
Radiation type	Mo *K*α	Mo *K*α
μ (mm^−1^)	0.24	0.37
Crystal size (mm)	0.40 × 0.10 × 0.05	0.15 × 0.15 × 0.10

Data collection		
Diffractometer	Bruker–Nonius Roper CCD camera on κ-goniostat	Bruker–Nonius APEXII CCD camera on κ-goniostat
Absorption correction	Multi-scan (*SADABS*; Sheldrick, 2007 ▸)	Multi-scan (*SADABS*; Sheldrick, 2007 ▸)
*T*_min_, *T*_max_	0.924, 1.000	0.974, 1.000
No. of measured, independent and observed [*I* > 2σ(*I*)] reflections	9476, 2519, 1833	24772, 2813, 2630
*R*_int_	0.067	0.034

Refinement		
*R*[*F* ^2^ > 2σ(*F* ^2^)], *wR*(*F* ^2^), *S*	0.045, 0.108, 1.04	0.038, 0.084, 1.14
No. of reflections	2519	2813
No. of parameters	170	192
No. of restraints	2	2
H-atom treatment	H atoms treated by a mixture of independent and constrained refinement	H atoms treated by a mixture of independent and constrained refinement
Δρ_max_, Δρ_min_ (e Å^−3^)	0.31, −0.27	0.53, −0.30

Computer programs: *DENZO* (Otwinowski & Minor, 1997 ▸), *COLLECT* (Hooft, 1998 ▸), *SHELXT* (Sheldrick, 2015*a* ▸), *SHELXL2014* (Sheldrick, 2015*b* ▸), *XP* in *SHELXTL* (Sheldrick, 2008 ▸) *Mercury* (Macrae *et al.*, 2006 ▸), *PLATON* (Spek, 2009 ▸), *publCIF* (Westrip, 2010 ▸) and *TOPOS* (Blatov, 2006 ▸).

## supplementary crystallographic information

### 5-(2-Methylpropyl)-5-(prop-2-en-1-yl)-2-sulfanylidene-1,3-diazinane-4,6-dione (I) Crystal data

**Table:**

C_11_H_16_N_2_O_2_S	*F*(000) = 512
*M**_r_* = 240.32	*D*_x_ = 1.260 Mg m^−^^3^
Monoclinic, *P*2_1_/*n*	Mo *K*α radiation, λ = 0.71073 Å
*a* = 8.7271 (6) Å	Cell parameters from 10435 reflections
*b* = 11.6521 (4) Å	θ = 2.9–27.5°
*c* = 12.5400 (8) Å	µ = 0.24 mm^−^^1^
β = 96.539 (2)°	*T* = 120 K
*V* = 1266.89 (13) Å^3^	Prism, colourless
*Z* = 4	0.40 × 0.10 × 0.05 mm

### 5-(2-Methylpropyl)-5-(prop-2-en-1-yl)-2-sulfanylidene-1,3-diazinane-4,6-dione (I) Data collection

**Table:**

Bruker–Nonius Roper CCD camera on κ-goniostat diffractometer	2519 independent reflections
Radiation source: Bruker-Nonius FR591 rotating anode	1833 reflections with *I* > 2σ(*I*)
Graphite monochromator	*R*_int_ = 0.067
Detector resolution: 9.091 pixels mm^-1^	θ_max_ = 26.4°, θ_min_ = 3.3°
φ & ω scans	*h* = −9→10
Absorption correction: multi-scan (SADABS; Sheldrick, 2007)	*k* = −12→13
*T*_min_ = 0.924, *T*_max_ = 1.000	*l* = −15→14
9476 measured reflections	

### 5-(2-Methylpropyl)-5-(prop-2-en-1-yl)-2-sulfanylidene-1,3-diazinane-4,6-dione (I) Refinement

**Table:**

Refinement on *F*^2^	Secondary atom site location: difference Fourier map
Least-squares matrix: full	Hydrogen site location: mixed
*R*[*F*^2^ > 2σ(*F*^2^)] = 0.045	H atoms treated by a mixture of independent and constrained refinement
*wR*(*F*^2^) = 0.108	*w* = 1/[σ^2^(*F*_o_^2^) + (0.0431*P*)^2^ + 0.281*P*] where *P* = (*F*_o_^2^ + 2*F*_c_^2^)/3
*S* = 1.04	(Δ/σ)_max_ < 0.001
2519 reflections	Δρ_max_ = 0.31 e Å^−^^3^
170 parameters	Δρ_min_ = −0.27 e Å^−^^3^
2 restraints	Extinction correction: SHELXL2014 (Sheldrick, 2015b), Fc^*^=kFc[1+0.001xFc^2^λ^3^/sin(2θ)]^-1/4^
Primary atom site location: structure-invariant direct methods	Extinction coefficient: 0.011 (2)

### 5-(2-Methylpropyl)-5-(prop-2-en-1-yl)-2-sulfanylidene-1,3-diazinane-4,6-dione (I) Special details

**Table:**

Geometry. All esds (except the esd in the dihedral angle between two l.s. planes) are estimated using the full covariance matrix. The cell esds are taken into account individually in the estimation of esds in distances, angles and torsion angles; correlations between esds in cell parameters are only used when they are defined by crystal symmetry. An approximate (isotropic) treatment of cell esds is used for estimating esds involving l.s. planes.

### 5-(2-Methylpropyl)-5-(prop-2-en-1-yl)-2-sulfanylidene-1,3-diazinane-4,6-dione (I) Fractional atomic coordinates and isotropic or equivalent isotropic displacement parameters (Å^2^)

**Table:**

	*x*	*y*	*z*	*U*_iso_*/*U*_eq_	
S2	0.13456 (7)	0.87633 (4)	0.94050 (5)	0.0259 (2)	
O4	0.42805 (18)	0.66380 (11)	0.69496 (13)	0.0256 (4)	
O6	0.26955 (18)	1.04475 (11)	0.60757 (12)	0.0261 (4)	
N1	0.2119 (2)	0.95525 (14)	0.75707 (14)	0.0197 (4)	
H1	0.162 (3)	1.0169 (17)	0.771 (2)	0.036 (7)*	
N3	0.2876 (2)	0.76850 (14)	0.79944 (14)	0.0192 (4)	
H3	0.282 (3)	0.7101 (16)	0.8409 (17)	0.030 (7)*	
C2	0.2138 (2)	0.86637 (16)	0.82860 (17)	0.0181 (5)	
C4	0.3650 (2)	0.75482 (16)	0.71139 (17)	0.0196 (5)	
C5	0.3698 (2)	0.85445 (16)	0.63413 (17)	0.0187 (5)	
C6	0.2810 (2)	0.95949 (16)	0.66426 (17)	0.0193 (5)	
C7	0.2939 (3)	0.81243 (17)	0.52224 (17)	0.0225 (5)	
H7A	0.3022	0.8740	0.4689	0.034 (7)*	
H7B	0.3518	0.7451	0.5000	0.034 (7)*	
C8	0.1275 (3)	0.7801 (2)	0.52146 (18)	0.0286 (5)	
H8	0.0538	0.8398	0.5236	0.038 (7)*	
C9	0.0781 (3)	0.6733 (2)	0.5180 (2)	0.0397 (7)	
H9A	0.1494	0.6119	0.5157	0.054 (9)*	
H9B	−0.0287	0.6576	0.5176	0.051 (8)*	
C10	0.5409 (3)	0.88428 (17)	0.62406 (17)	0.0221 (5)	
H10A	0.5867	0.8189	0.5884	0.029 (6)*	
H10B	0.5423	0.9512	0.5756	0.025 (6)*	
C11	0.6456 (3)	0.9114 (2)	0.7264 (2)	0.0322 (6)	
H11	0.6314	0.8501	0.7802	0.052 (8)*	
C12	0.6132 (3)	1.0262 (3)	0.7760 (2)	0.0494 (8)	
H12A	0.6195	1.0872	0.7228	0.074 (11)*	
H12B	0.6895	1.0404	0.8382	0.076 (10)*	
H12C	0.5096	1.0254	0.7990	0.074 (11)*	
C13	0.8130 (3)	0.9075 (3)	0.7015 (3)	0.0484 (8)	
H13A	0.8366	0.8305	0.6764	0.068 (10)*	
H13B	0.8819	0.9256	0.7666	0.069 (10)*	
H13C	0.8278	0.9639	0.6456	0.049 (8)*	

### 5-(2-Methylpropyl)-5-(prop-2-en-1-yl)-2-sulfanylidene-1,3-diazinane-4,6-dione (I) Atomic displacement parameters (Å^2^)

**Table:**

	*U*^11^	*U*^22^	*U*^33^	*U*^12^	*U*^13^	*U*^23^
S2	0.0311 (4)	0.0251 (3)	0.0237 (3)	0.0012 (2)	0.0123 (3)	0.0027 (2)
O4	0.0280 (9)	0.0144 (7)	0.0365 (10)	0.0023 (6)	0.0129 (7)	0.0001 (6)
O6	0.0392 (10)	0.0170 (8)	0.0238 (9)	0.0019 (6)	0.0113 (7)	0.0035 (6)
N1	0.0239 (11)	0.0145 (9)	0.0222 (10)	0.0010 (7)	0.0086 (8)	0.0010 (7)
N3	0.0234 (10)	0.0139 (9)	0.0212 (10)	0.0015 (7)	0.0063 (8)	0.0034 (7)
C2	0.0164 (11)	0.0172 (10)	0.0203 (11)	−0.0026 (8)	0.0002 (9)	−0.0009 (8)
C4	0.0164 (11)	0.0178 (11)	0.0248 (12)	−0.0042 (8)	0.0031 (9)	−0.0019 (8)
C5	0.0214 (12)	0.0154 (10)	0.0205 (11)	−0.0015 (8)	0.0074 (9)	−0.0004 (8)
C6	0.0222 (12)	0.0160 (10)	0.0200 (12)	−0.0020 (8)	0.0042 (9)	−0.0018 (8)
C7	0.0262 (13)	0.0213 (11)	0.0207 (12)	−0.0024 (9)	0.0063 (10)	−0.0025 (8)
C8	0.0259 (13)	0.0358 (13)	0.0240 (13)	−0.0013 (10)	0.0028 (10)	−0.0040 (10)
C9	0.0355 (16)	0.0477 (16)	0.0343 (15)	−0.0172 (13)	−0.0032 (12)	0.0032 (11)
C10	0.0238 (12)	0.0215 (11)	0.0225 (12)	−0.0024 (8)	0.0088 (10)	0.0005 (8)
C11	0.0269 (14)	0.0405 (14)	0.0287 (14)	−0.0073 (10)	0.0016 (11)	0.0039 (10)
C12	0.0388 (18)	0.068 (2)	0.0405 (17)	−0.0152 (14)	0.0031 (14)	−0.0245 (15)
C13	0.0291 (16)	0.066 (2)	0.0496 (19)	−0.0035 (13)	0.0025 (14)	0.0078 (15)

### 5-(2-Methylpropyl)-5-(prop-2-en-1-yl)-2-sulfanylidene-1,3-diazinane-4,6-dione (I) Geometric parameters (Å, º)

**Table:**

S2—C2	1.638 (2)	C8—C9	1.315 (3)
O4—C4	1.223 (2)	C8—H8	0.9500
O6—C6	1.219 (2)	C9—H9A	0.9500
N1—C2	1.369 (3)	C9—H9B	0.9500
N1—C6	1.371 (3)	C10—C11	1.522 (3)
N1—H1	0.867 (16)	C10—H10A	0.9900
N3—C4	1.367 (3)	C10—H10B	0.9900
N3—C2	1.379 (3)	C11—C12	1.515 (4)
N3—H3	0.861 (16)	C11—C13	1.529 (4)
C4—C5	1.516 (3)	C11—H11	1.0000
C5—C6	1.519 (3)	C12—H12A	0.9800
C5—C10	1.552 (3)	C12—H12B	0.9800
C5—C7	1.561 (3)	C12—H12C	0.9800
C7—C8	1.499 (3)	C13—H13A	0.9800
C7—H7A	0.9900	C13—H13B	0.9800
C7—H7B	0.9900	C13—H13C	0.9800
			
C2—N1—C6	127.57 (17)	C7—C8—H8	118.3
C2—N1—H1	117.7 (17)	C8—C9—H9A	120.0
C6—N1—H1	114.8 (17)	C8—C9—H9B	120.0
C4—N3—C2	126.79 (17)	H9A—C9—H9B	120.0
C4—N3—H3	117.7 (16)	C11—C10—C5	117.95 (18)
C2—N3—H3	115.5 (16)	C11—C10—H10A	107.8
N1—C2—N3	115.02 (18)	C5—C10—H10A	107.8
N1—C2—S2	122.21 (15)	C11—C10—H10B	107.8
N3—C2—S2	122.77 (15)	C5—C10—H10B	107.8
O4—C4—N3	120.68 (18)	H10A—C10—H10B	107.2
O4—C4—C5	120.70 (19)	C12—C11—C10	114.0 (2)
N3—C4—C5	118.62 (17)	C12—C11—C13	109.7 (2)
C4—C5—C6	113.95 (17)	C10—C11—C13	108.5 (2)
C4—C5—C10	108.69 (17)	C12—C11—H11	108.1
C6—C5—C10	111.26 (16)	C10—C11—H11	108.1
C4—C5—C7	107.13 (16)	C13—C11—H11	108.1
C6—C5—C7	107.46 (17)	C11—C12—H12A	109.5
C10—C5—C7	108.11 (16)	C11—C12—H12B	109.5
O6—C6—N1	120.64 (18)	H12A—C12—H12B	109.5
O6—C6—C5	121.44 (18)	C11—C12—H12C	109.5
N1—C6—C5	117.91 (17)	H12A—C12—H12C	109.5
C8—C7—C5	113.35 (17)	H12B—C12—H12C	109.5
C8—C7—H7A	108.9	C11—C13—H13A	109.5
C5—C7—H7A	108.9	C11—C13—H13B	109.5
C8—C7—H7B	108.9	H13A—C13—H13B	109.5
C5—C7—H7B	108.9	C11—C13—H13C	109.5
H7A—C7—H7B	107.7	H13A—C13—H13C	109.5
C9—C8—C7	123.4 (2)	H13B—C13—H13C	109.5
C9—C8—H8	118.3		
			
C6—N1—C2—N3	−3.7 (3)	C10—C5—C6—O6	59.5 (3)
C6—N1—C2—S2	176.16 (17)	C7—C5—C6—O6	−58.7 (2)
C4—N3—C2—N1	4.2 (3)	C4—C5—C6—N1	2.0 (3)
C4—N3—C2—S2	−175.64 (17)	C10—C5—C6—N1	−121.3 (2)
C2—N3—C4—O4	178.9 (2)	C7—C5—C6—N1	120.6 (2)
C2—N3—C4—C5	−1.6 (3)	C4—C5—C7—C8	62.7 (2)
O4—C4—C5—C6	177.91 (19)	C6—C5—C7—C8	−60.1 (2)
N3—C4—C5—C6	−1.6 (3)	C10—C5—C7—C8	179.67 (17)
O4—C4—C5—C10	−57.4 (2)	C5—C7—C8—C9	−106.1 (3)
N3—C4—C5—C10	123.1 (2)	C4—C5—C10—C11	−55.6 (2)
O4—C4—C5—C7	59.2 (3)	C6—C5—C10—C11	70.6 (2)
N3—C4—C5—C7	−120.3 (2)	C7—C5—C10—C11	−171.58 (18)
C2—N1—C6—O6	179.9 (2)	C5—C10—C11—C12	−71.7 (3)
C2—N1—C6—C5	0.6 (3)	C5—C10—C11—C13	165.64 (19)
C4—C5—C6—O6	−177.20 (19)		

### 5-(2-Methylpropyl)-5-(prop-2-en-1-yl)-2-sulfanylidene-1,3-diazinane-4,6-dione (I) Hydrogen-bond geometry (Å, º)

**Table:**

*D*—H···*A*	*D*—H	H···*A*	*D*···*A*	*D*—H···*A*
N1—H1···O4^i^	0.87 (2)	1.95 (2)	2.815 (2)	174 (2)
N3—H3···O6^ii^	0.86 (2)	2.10 (2)	2.922 (2)	160 (2)

Symmetry codes: (i) −*x*+1/2, *y*+1/2, −*z*+3/2; (ii) −*x*+1/2, *y*−1/2, −*z*+3/2.

### 5-(1-Methylbutyl)-5-[2-(methylsulfanyl)ethyl]-2-sulfanylidene-1,3-diazinane-4,6-dione (II) Crystal data

**Table:**

C_12_H_20_N_2_O_2_S_2_	*F*(000) = 1232
*M**_r_* = 288.42	*D*_x_ = 1.337 Mg m^−^^3^
Monoclinic, *C*2/*c*	Mo *K*α radiation, λ = 0.71073 Å
*a* = 15.1873 (2) Å	Cell parameters from 29667 reflections
*b* = 9.0920 (1) Å	θ = 2.9–27.5°
*c* = 20.8684 (3) Å	µ = 0.37 mm^−^^1^
β = 96.083 (1)°	*T* = 120 K
*V* = 2865.34 (6) Å^3^	Block, colourless
*Z* = 8	0.15 × 0.15 × 0.10 mm

### 5-(1-Methylbutyl)-5-[2-(methylsulfanyl)ethyl]-2-sulfanylidene-1,3-diazinane-4,6-dione (II) Data collection

**Table:**

Bruker–Nonius APEXII CCD camera on κ-goniostat diffractometer	2813 independent reflections
Radiation source: Bruker-Nonius FR591 rotating anode	2630 reflections with *I* > 2σ(*I*)
10cm confocal mirrors monochromator	*R*_int_ = 0.034
Detector resolution: 9.091 pixels mm^-1^	θ_max_ = 26.0°, θ_min_ = 3.2°
φ & ω scans	*h* = −18→18
Absorption correction: multi-scan (SADABS; Sheldrick, 2007)	*k* = −11→11
*T*_min_ = 0.974, *T*_max_ = 1.000	*l* = −25→25
24772 measured reflections	

### 5-(1-Methylbutyl)-5-[2-(methylsulfanyl)ethyl]-2-sulfanylidene-1,3-diazinane-4,6-dione (II) Refinement

**Table:**

Refinement on *F*^2^	2 restraints
Least-squares matrix: full	Hydrogen site location: mixed
*R*[*F*^2^ > 2σ(*F*^2^)] = 0.038	H atoms treated by a mixture of independent and constrained refinement
*wR*(*F*^2^) = 0.084	*w* = 1/[σ^2^(*F*_o_^2^) + (0.0192*P*)^2^ + 6.3729*P*] where *P* = (*F*_o_^2^ + 2*F*_c_^2^)/3
*S* = 1.14	(Δ/σ)_max_ = 0.001
2813 reflections	Δρ_max_ = 0.53 e Å^−^^3^
192 parameters	Δρ_min_ = −0.29 e Å^−^^3^

### 5-(1-Methylbutyl)-5-[2-(methylsulfanyl)ethyl]-2-sulfanylidene-1,3-diazinane-4,6-dione (II) Special details

**Table:**

Geometry. All esds (except the esd in the dihedral angle between two l.s. planes) are estimated using the full covariance matrix. The cell esds are taken into account individually in the estimation of esds in distances, angles and torsion angles; correlations between esds in cell parameters are only used when they are defined by crystal symmetry. An approximate (isotropic) treatment of cell esds is used for estimating esds involving l.s. planes.

### 5-(1-Methylbutyl)-5-[2-(methylsulfanyl)ethyl]-2-sulfanylidene-1,3-diazinane-4,6-dione (II) Fractional atomic coordinates and isotropic or equivalent isotropic displacement parameters (Å^2^)

**Table:**

	*x*	*y*	*z*	*U*_iso_*/*U*_eq_	
S2	−0.17769 (3)	0.62565 (6)	0.34779 (2)	0.02411 (13)	
S9	0.14236 (3)	1.10685 (5)	0.51192 (2)	0.02392 (13)	
O4	0.09157 (9)	0.87624 (15)	0.30012 (6)	0.0218 (3)	
O6	0.09747 (9)	0.59147 (14)	0.49086 (6)	0.0220 (3)	
N1	−0.02312 (10)	0.60656 (17)	0.41868 (7)	0.0177 (3)	
H1	−0.0510 (14)	0.551 (2)	0.4430 (10)	0.031 (6)*	
N3	−0.02774 (10)	0.75738 (17)	0.32870 (7)	0.0181 (3)	
H3	−0.0574 (14)	0.798 (2)	0.2962 (9)	0.029 (6)*	
C2	−0.07264 (12)	0.6645 (2)	0.36566 (8)	0.0173 (4)	
C4	0.06055 (12)	0.79499 (19)	0.33818 (8)	0.0167 (4)	
C6	0.06356 (12)	0.63907 (19)	0.43924 (8)	0.0171 (4)	
C7	0.16068 (12)	0.85980 (19)	0.43567 (8)	0.0161 (4)	
H7A	0.2088	0.8208	0.4667	0.020 (5)*	
H7B	0.1875	0.9281	0.4063	0.021 (5)*	
C5	0.11666 (11)	0.73082 (19)	0.39593 (8)	0.0158 (4)	
C8	0.09525 (13)	0.9443 (2)	0.47235 (9)	0.0218 (4)	
H8A	0.0735	0.8786	0.5051	0.029 (6)*	
H8B	0.0437	0.9733	0.4419	0.031 (6)*	
C10	0.13117 (16)	1.2337 (2)	0.44571 (12)	0.0345 (5)	
H10A	0.1605	1.1935	0.4099	0.071 (10)*	
H10B	0.1586	1.3277	0.4594	0.053 (8)*	
H10C	0.0682	1.2492	0.4316	0.047 (8)*	
C12	0.18817 (13)	0.6231 (2)	0.37204 (9)	0.0217 (4)	
H12	0.2155	0.5694	0.4110	0.033 (6)*	
C13	0.26315 (13)	0.7017 (2)	0.34349 (10)	0.0258 (4)	
H13A	0.2880	0.7777	0.3742	0.040 (7)*	
H13B	0.2390	0.7523	0.3034	0.043 (7)*	
C14	0.33787 (14)	0.5984 (2)	0.32810 (11)	0.0308 (5)	
H14A	0.3564	0.5368	0.3662	0.029 (6)*	
H14B	0.3159	0.5324	0.2922	0.054 (8)*	
C15	0.41659 (17)	0.6846 (3)	0.30960 (13)	0.0438 (6)	
H15A	0.3988	0.7425	0.2708	0.072 (10)*	
H15B	0.4640	0.6164	0.3011	0.053 (8)*	
H15C	0.4380	0.7508	0.3449	0.073 (10)*	
C16	0.14371 (14)	0.5061 (2)	0.32611 (9)	0.0250 (4)	
H16A	0.1226	0.5521	0.2849	0.036 (7)*	
H16B	0.0935	0.4628	0.3452	0.038 (7)*	
H16C	0.1867	0.4290	0.3190	0.040 (7)*	

### 5-(1-Methylbutyl)-5-[2-(methylsulfanyl)ethyl]-2-sulfanylidene-1,3-diazinane-4,6-dione (II) Atomic displacement parameters (Å^2^)

**Table:**

	*U*^11^	*U*^22^	*U*^33^	*U*^12^	*U*^13^	*U*^23^
S2	0.0188 (2)	0.0307 (3)	0.0228 (2)	−0.0057 (2)	0.00256 (18)	0.0002 (2)
S9	0.0251 (3)	0.0196 (2)	0.0270 (3)	−0.00207 (19)	0.00215 (19)	−0.00781 (19)
O4	0.0228 (7)	0.0243 (7)	0.0184 (6)	−0.0061 (6)	0.0029 (5)	0.0052 (5)
O6	0.0248 (7)	0.0210 (7)	0.0195 (7)	−0.0020 (6)	−0.0006 (5)	0.0056 (5)
N1	0.0194 (8)	0.0159 (7)	0.0183 (8)	−0.0032 (6)	0.0045 (6)	0.0025 (6)
N3	0.0177 (8)	0.0212 (8)	0.0152 (7)	−0.0008 (6)	0.0009 (6)	0.0030 (6)
C2	0.0211 (9)	0.0159 (9)	0.0152 (8)	−0.0005 (7)	0.0038 (7)	−0.0027 (7)
C4	0.0200 (9)	0.0157 (9)	0.0145 (8)	0.0003 (7)	0.0028 (7)	−0.0030 (7)
C6	0.0209 (9)	0.0119 (8)	0.0186 (9)	−0.0001 (7)	0.0029 (7)	−0.0018 (7)
C7	0.0164 (9)	0.0147 (8)	0.0172 (8)	−0.0012 (7)	0.0023 (7)	−0.0011 (7)
C5	0.0153 (8)	0.0155 (8)	0.0169 (8)	0.0004 (7)	0.0026 (7)	0.0000 (7)
C8	0.0230 (10)	0.0169 (9)	0.0266 (10)	−0.0043 (8)	0.0081 (8)	−0.0067 (8)
C10	0.0388 (13)	0.0191 (10)	0.0469 (13)	0.0002 (9)	0.0106 (11)	0.0060 (10)
C12	0.0222 (10)	0.0183 (9)	0.0250 (10)	0.0029 (8)	0.0047 (8)	−0.0032 (8)
C13	0.0249 (10)	0.0237 (10)	0.0299 (10)	0.0025 (8)	0.0081 (8)	0.0006 (8)
C14	0.0283 (11)	0.0317 (12)	0.0345 (12)	0.0080 (9)	0.0122 (9)	−0.0006 (9)
C15	0.0387 (14)	0.0430 (14)	0.0528 (15)	0.0091 (12)	0.0189 (12)	0.0070 (13)
C16	0.0312 (11)	0.0189 (9)	0.0258 (10)	0.0007 (8)	0.0078 (8)	−0.0048 (8)

### 5-(1-Methylbutyl)-5-[2-(methylsulfanyl)ethyl]-2-sulfanylidene-1,3-diazinane-4,6-dione (II) Geometric parameters (Å, º)

**Table:**

S2—C2	1.6381 (19)	C8—H8B	0.9900
S9—C10	1.794 (2)	C10—H10A	0.9800
S9—C8	1.8033 (19)	C10—H10B	0.9800
O4—C4	1.216 (2)	C10—H10C	0.9800
O6—C6	1.223 (2)	C12—C13	1.519 (3)
N1—C6	1.373 (2)	C12—C16	1.539 (3)
N1—C2	1.375 (2)	C12—H12	1.0000
N1—H1	0.858 (16)	C13—C14	1.533 (3)
N3—C2	1.373 (2)	C13—H13A	0.9900
N3—C4	1.378 (2)	C13—H13B	0.9900
N3—H3	0.856 (16)	C14—C15	1.513 (3)
C4—C5	1.517 (2)	C14—H14A	0.9900
C6—C5	1.522 (2)	C14—H14B	0.9900
C7—C8	1.525 (2)	C15—H15A	0.9800
C7—C5	1.547 (2)	C15—H15B	0.9800
C7—H7A	0.9900	C15—H15C	0.9800
C7—H7B	0.9900	C16—H16A	0.9800
C5—C12	1.582 (2)	C16—H16B	0.9800
C8—H8A	0.9900	C16—H16C	0.9800
			
C10—S9—C8	99.99 (10)	S9—C10—H10B	109.5
C6—N1—C2	126.40 (15)	H10A—C10—H10B	109.5
C6—N1—H1	117.3 (16)	S9—C10—H10C	109.5
C2—N1—H1	116.1 (16)	H10A—C10—H10C	109.5
C2—N3—C4	127.29 (16)	H10B—C10—H10C	109.5
C2—N3—H3	117.3 (16)	C13—C12—C16	112.25 (16)
C4—N3—H3	115.4 (16)	C13—C12—C5	113.62 (15)
N3—C2—N1	115.21 (16)	C16—C12—C5	110.71 (15)
N3—C2—S2	122.31 (14)	C13—C12—H12	106.6
N1—C2—S2	122.47 (14)	C16—C12—H12	106.6
O4—C4—N3	119.72 (16)	C5—C12—H12	106.6
O4—C4—C5	121.92 (16)	C12—C13—C14	113.35 (17)
N3—C4—C5	118.36 (15)	C12—C13—H13A	108.9
O6—C6—N1	119.97 (16)	C14—C13—H13A	108.9
O6—C6—C5	121.12 (16)	C12—C13—H13B	108.9
N1—C6—C5	118.89 (15)	C14—C13—H13B	108.9
C8—C7—C5	112.56 (14)	H13A—C13—H13B	107.7
C8—C7—H7A	109.1	C15—C14—C13	111.01 (19)
C5—C7—H7A	109.1	C15—C14—H14A	109.4
C8—C7—H7B	109.1	C13—C14—H14A	109.4
C5—C7—H7B	109.1	C15—C14—H14B	109.4
H7A—C7—H7B	107.8	C13—C14—H14B	109.4
C4—C5—C6	113.21 (15)	H14A—C14—H14B	108.0
C4—C5—C7	107.99 (14)	C14—C15—H15A	109.5
C6—C5—C7	108.86 (14)	C14—C15—H15B	109.5
C4—C5—C12	109.53 (14)	H15A—C15—H15B	109.5
C6—C5—C12	105.81 (14)	C14—C15—H15C	109.5
C7—C5—C12	111.49 (14)	H15A—C15—H15C	109.5
C7—C8—S9	113.25 (13)	H15B—C15—H15C	109.5
C7—C8—H8A	108.9	C12—C16—H16A	109.5
S9—C8—H8A	108.9	C12—C16—H16B	109.5
C7—C8—H8B	108.9	H16A—C16—H16B	109.5
S9—C8—H8B	108.9	C12—C16—H16C	109.5
H8A—C8—H8B	107.7	H16A—C16—H16C	109.5
S9—C10—H10A	109.5	H16B—C16—H16C	109.5
			
C4—N3—C2—N1	1.7 (3)	N1—C6—C5—C7	128.90 (16)
C4—N3—C2—S2	−178.33 (14)	O6—C6—C5—C12	67.0 (2)
C6—N1—C2—N3	3.8 (3)	N1—C6—C5—C12	−111.17 (17)
C6—N1—C2—S2	−176.19 (14)	C8—C7—C5—C4	73.15 (18)
C2—N3—C4—O4	178.09 (17)	C8—C7—C5—C6	−50.13 (19)
C2—N3—C4—C5	−1.0 (3)	C8—C7—C5—C12	−166.48 (15)
C2—N1—C6—O6	172.51 (17)	C5—C7—C8—S9	−173.66 (12)
C2—N1—C6—C5	−9.3 (3)	C10—S9—C8—C7	82.54 (16)
O4—C4—C5—C6	176.79 (16)	C4—C5—C12—C13	73.5 (2)
N3—C4—C5—C6	−4.2 (2)	C6—C5—C12—C13	−164.15 (16)
O4—C4—C5—C7	56.2 (2)	C7—C5—C12—C13	−46.0 (2)
N3—C4—C5—C7	−124.77 (16)	C4—C5—C12—C16	−53.9 (2)
O4—C4—C5—C12	−65.4 (2)	C6—C5—C12—C16	68.47 (19)
N3—C4—C5—C12	113.64 (17)	C7—C5—C12—C16	−173.34 (15)
O6—C6—C5—C4	−173.00 (16)	C16—C12—C13—C14	−60.5 (2)
N1—C6—C5—C4	8.8 (2)	C5—C12—C13—C14	172.91 (17)
O6—C6—C5—C7	−52.9 (2)	C12—C13—C14—C15	−171.28 (19)

### 5-(1-Methylbutyl)-5-[2-(methylsulfanyl)ethyl]-2-sulfanylidene-1,3-diazinane-4,6-dione (II) Hydrogen-bond geometry (Å, º)

**Table:**

*D*—H···*A*	*D*—H	H···*A*	*D*···*A*	*D*—H···*A*
N1—H1···O6^i^	0.86 (2)	2.07 (2)	2.921 (2)	170 (2)
N3—H3···O4^ii^	0.86 (2)	2.14 (2)	2.963 (2)	160 (2)

Symmetry codes: (i) −*x*, −*y*+1, −*z*+1; (ii) −*x*, *y*, −*z*+1/2.
