# Supplementary material for: Buthalital and methitural – 5,5-substituted derivatives of 2-thio­barbituric acid forming the same type of hydrogen-bonded chain
Source: Acta Crystallogr E Crystallogr Commun. 2017 Nov 21;73(Pt 12):1908–12. doi: 10.1107/S205698901701653X (PMC5730250; doi:10.1107/S205698901701653X)
Supplement: Supplementary file 6 [file e-73-01908-sup6.pdf]

supporting information

Buthalital and methitural – 5,5-substituted derivatives of 2-thiobarbituric acid forming the same type of H-bonded chain

Thomas Gelbrich and Ulrich J. Griesser*

Hot-stage microscopy of the

Experimental. An Olympus BH2 polarisation microscope (Olympus Optical GmbH, Vienna, Austria) equipped with a Kofler hot stage (Reichert Thermovar, Vienna, Austria) was used and images were recorded with an Olympus DP70 digital camera in connection with the cellD software (Soft Imaging System, Hamburg, Germany).

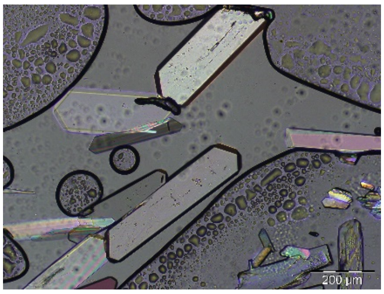

Fig. S1 Melt and crystals of form I of buthalital at 144 °C.

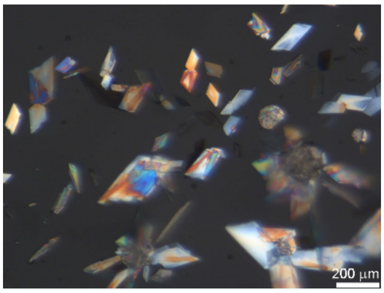

Fig. S2 Crystals of form II of buthalital, obtained by annealing the melt in silicon oil on a glass slide at 60 °C on a hot bench for 15 min.

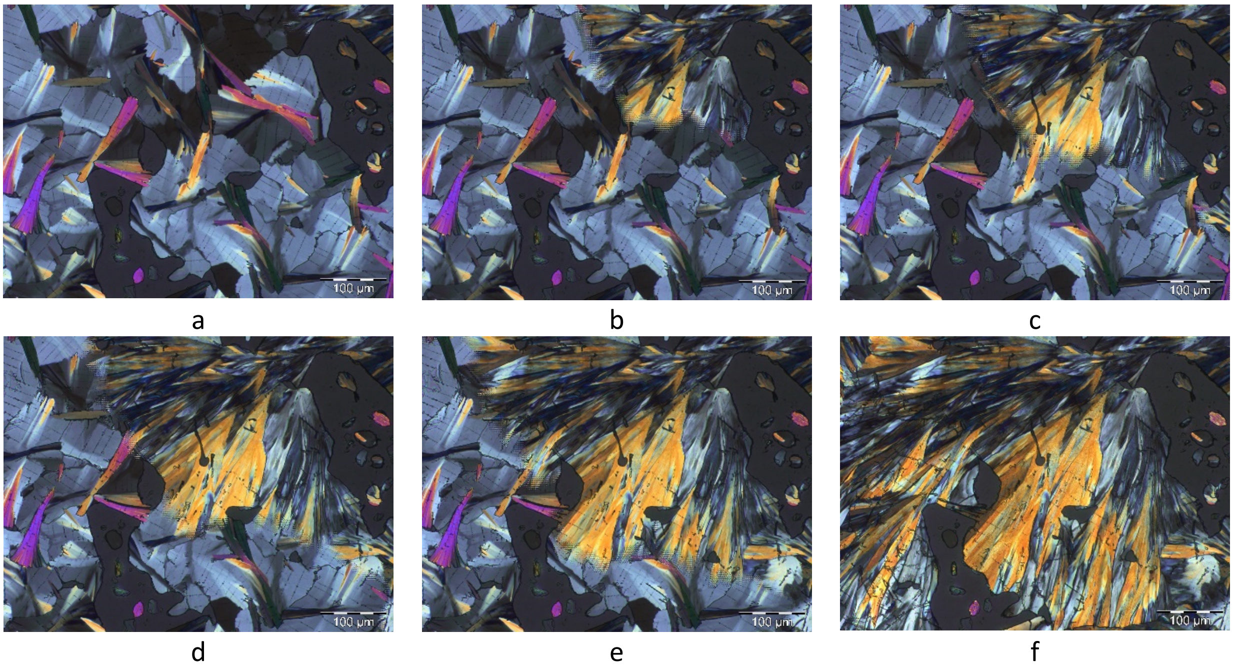

Fig. S2 Transformation of form II of buthalital into form I on heating between 105 and 110 °C: a) form II, obtained by heating the quench-cooled melt; b)-e) sequential stages of the phase transition between 105 and 110 °C; f) form I after the transformation is largely complete.
